# Fourier Transform Infrared Spectroscopic Characterization of Aortic Wall Remodeling by Stable Gastric Pentadecapeptide BPC 157 After Unilateral Adrenalectomy in Rats

**DOI:** 10.3390/ph19010191

**Published:** 2026-01-22

**Authors:** Ivan Maria Smoday, Vlasta Vukovic, Katarina Oroz, Hrvoje Vranes, Luka Kalogjera, Ozren Gamulin, Josipa Vlainic, Marija Milavic, Suncana Sikiric, Nora Nikolac Gabaj, Domagoj Marijancevic, Antun Koprivanac, Lidija Beketic Oreskovic, Ivana Oreskovic, Sanja Strbe, Ivan Barisic, Mario Kordic, Ante Tvrdeic, Sven Seiwerth, Predrag Sikiric, Alenka Boban Blagaic, Anita Skrtic

**Affiliations:** 1 Department of Pharmacology, School of Medicine, University of Zagreb, 10000 Zagreb, Croatia; 2 Department of Physics and Biophysics, School of Medicine, University of Zagreb, 10000 Zagreb, Croatia; 3 Laboratory for Advanced Genomics, Division of Molecular Medicine, Institute Ruder Boskovic, 10000 Zagreb, Croatia; josipa.vlainic@irb.hr; 4 Department of Pathology, School of Medicine, University of Zagreb, 10000 Zagreb, Croatia; 5 Department of Chemistry, University Clinical Hospital Center “Sestre Milosrdnice”, 10000 Zagreb, Croatia

**Keywords:** FTIR spectroscopy, unilateral adrenalectomy, BPC 157, aortic wall remodeling

## Abstract

**Background:** No Fourier transform infrared (FTIR) spectroscopy studies have directly evaluated adrenalectomy vessels, the technique’s established ability to probe collagen/elastin-associated spectral features and lipid peroxidation-related signatures, and protein structural damage. Stable gastric pentadecapeptide BPC 157 therapy was found to maintain the vascular function under severe stress, as FTIR spectroscopy recently demonstrated rapid peptide-induced molecular changes in healthy rat blood vessels, particularly in lipid content and protein secondary structure. **Methods:** To extend these findings and highlight the BPC 157 vascular background in the special circumstances of the course following unilateral adrenalectomy, abdominal aortas were collected at 15 min, 5 h, and 24 h after unilateral adrenalectomy for the FTIR spectroscopy assessment. **Results:** FTIR spectra were acquired, preprocessed, and analyzed using principal component analysis (PCA), support vector machine discriminant analysis (SVMDA), and band-specific statistics. BPC 157 (10 ng/kg intragatrically immediately after unilateral adrenalectomy) produced a clear, reproducible separation of aortic spectra from control samples at all time points. The main discriminatory spectral signatures were observed in three regions, including amide I and amide II (protein-related bands, consistent with collagen/elastin contributions) and lipid C–H stretching bands. These spectral signatures are consistent with early extracellular matrix reinforcement and membrane preservation in the vascular wall and align with the recovering effect on the lesions in counteraction of the severe vascular and multiorgan failure, attenuation/elimination of thrombosis and blood pressure disturbances in various occlusion/occlusion-like syndromes. **Conclusions:** Together, after unilateral adrenalectomy, the FTIR data provide molecular-level spectral signatures consistent with rapid remodeling of the aortic wall toward a more structurally stable and functionally favorable state.

## 1. Introduction

This study focused on the presentation of blood vessels, aortic wall remodeling in rats after unilateral adrenalectomy, assessed by Fourier Transform Infrared (FTIR) spectroscopy [1], and the effect of the stable gastric pentadecapeptide BPC 157 as a therapy [1,2].

As the adrenal gland is commonly acknowledged as of the highest importance, the adrenalectomy is one of the most investigated topics [3,4,5,6,7]. However, the special issue is the vascular fragility or related hemodynamic effects after adrenalectomy [8,9,10,11,12,13]. In particular, adrenalectomized animals lacked cytoprotective capabilities and failed to exhibit the typical mucosal protective response [14]. Conceptually, combined with the adrenal gland, function and dysfunction [14], the long-ago established theory, cytoprotection theory, which originated in fundamental papers on stomach protection [15,16,17,18,19,20,21,22,23], holds that the spreading of the essential role of endothelium maintenance and recovery and vascular integrity carries cytoprotection capabilities into pleiotropic beneficial effects (cytoprotection→organoprotection) and occurs via application of cytoprotective agents [15,16,17,18,19,20,21,22,23]. Likewise, as a novel resolution, we focused on the stable gastric pentadecapeptide BPC 157’s special vascular recovery effect [1,2], already demonstrated in FTIR studies [1]; however, it is much more investigated in animal studies [1,2] than in clinical trials (ulcerative colitis (double-blind phase II) [24,25], recovery of knee pain, and intestinal cystitis (small studies) [26,27]). Very safe without adverse effects, lethal dose (LD1) not achieved in toxicology studies, likely acting as a cytoprotection mediator, maintaining stomach and gastrointestinal tract integrity, and as native and stable in human gastric juice for more than 24 h [1,2], BPC 157 was applied intragastrically, after unilateral adrenalectomy.

Its special vascular effect arose from its very early studies [28], implicating its innate cytoprotection role [1,2]. Recently, this vascular recovery was expanded to the activation of the collateral rescuing pathways [2]. As reviewed recently [2], this was shown to be essential for recovery of severe vascular and multiorgan failure in various occlusion/occlusion-like syndromes initially shown in rats with occluded major vessels (i.e., superior mesenteric vein, superior sagittal sinus) [29,30]. In addition, the severe intracranial, portal, and caval hypertension, aortal hypotension, hemorrhage, widespread thrombosis, and advanced Virchow triad circumstances, were regularly all attenuated or even eliminated [29,30]. Likewise, due to this vascular response [2], the severe lesions in the brain, heart, lung, liver, kidney, and gastrointestinal tract were attenuated [29,30]. Similar therapy beneficial effects and similar pathology occurred in rats subjected to severe, similar noxious procedures (i.e., increased abdominal pressure grade III and grade IV) [31,32], or different agents’ application (isoprenaline-myocardial infarction) [33] (and thereby, termed occlusion/occlusion-like syndrome) [2]. There, while interacting with various molecular pathways [34,35,36,37,38,39,40,41,42,43,44,45,46] (i.e., functioning as a stabilizer of cellular junction [42], leading to the significantly mitigated leaky gut syndrome) and NO-system, in particular [44,45,46], its pleiotropic beneficial effects are anchored to its resolving effects on increased angiogenesis, increased VEGF, increased egr-1 gene, increased NO, or eNOS stimulation, and increased free radical formation [2]. Consequently, BPC 157 was pointed out as a therapy and safety key: a special beneficial pleiotropic effect controlling and modulating angiogenesis and the NO-system [2]. As several BPC 157 studies demonstrated its free radical scavenger effects (see for review [2]), NO-level in tissue, increased or decreased, was regularly normalized through BPC 157 administration, along with a decrease (and/or normalization) of the increased malondialdehyde (MDA) level [2].

Notably, FTIR [1,47,48,49,50,51,52,53,54] is exceptionally well-suited to detect these changes quantitatively, early, and in a label-free manner. While no FTIR studies have directly evaluated adrenalectomy vessels, the technique’s established ability to measure collagen/elastin content, lipid peroxidation, and protein structural damage strongly supports its application.

Previously, our study [1] included healthy rats treated with BPC 157 (10 ng/kg, i.p.), harvested thoracic/abdominal aorta ~90 min later, and applied FTIR spectroscopy. FTIR + chemometric (principal component analysis, PCA) data revealed clear spectral particularities for BPC 157 therapy, the protein amide I band (~1650 cm^−1^), amide II band (~1540 cm^−1^), and a lipid-/ester-related band (~1744 cm^−1^). The less intense 1744 cm^−1^ band in BPC-treated rats was indicative of advanced early cell-death changes (non-hydrogen-bonded ester carbonyls in phospholipids) in controls with a more intense 1744 cm^−1^ band, and thereby, “protected” vascular tissue from early degenerative changes by BPC 157 therapy. Thus, the changes in protein secondary-structure conformation (as inferred from amide I/II band shifts), and alterations in lipid content were proposed to reflect a rapid cytoprotective effect of BPC 157 on vascular wall components [1].

Finally, to support BPC 157 therapy for the vascular function under severe stress [1], FTIR spectroscopy was recently introduced to demonstrate rapid peptide-induced molecular changes in healthy rat blood vessels, particularly in lipid content and protein secondary structure. Therefore, to extend these findings and highlight the BPC 157 vascular background, providing the special circumstances of the course following unilateral adrenalectomy, the 15 min, 5 h, and 24 h were used for the FTIR spectroscopy assessment. These intervals were also commonly assessed in the noted recovery of the various occlusion/occlusion-like syndromes [29,30,31,32,33]. Notably, this would be the first demonstration of FTIR-detectable extracellular matrix (ECM) and lipid recovery in adrenalectomized vasculature.

## 2. Results

### 2.1. Principal Component Analysis (PCA) and Support Vector Machine Discriminant Analysis (SVMDA) Reveal Separation of Aortic Spectra

**Figure 1:**
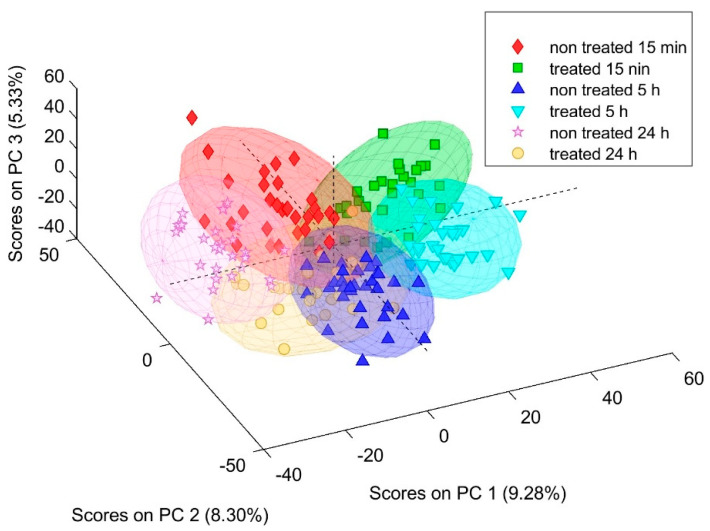
FTIR PCA 3D scores plot (PC1–PC2–PC3) of aortic spectra from control (non-treated (NT)) and BPC 157-treated (BPC) rats at 15 min, 5 h, and 24 h after unilateral adrenalectomy.

FTIR spectra of abdominal aorta sections from control and BPC 157-treated animals showed clear treatment- and time-dependent clustering in multivariate space. In the three-dimensional PCA score plot (PC1–PC2–PC3), six distinct clusters were observed, corresponding to the control and treated groups at 15 min, 5 h, and 24 h after adrenalectomy (Figure 1). The first three PCs together accounted for approximately one quarter of the total spectral variance, with PC1 explaining over 10%, PC2 around 7%, and PC3 about 6%. Despite partial overlap of individual spectra within each group, BPC 157-treated samples were consistently separated from saline controls along PC1 at all time points, indicating reproducible peptide-induced biochemical changes in the vascular wall.

**Figure 2:**
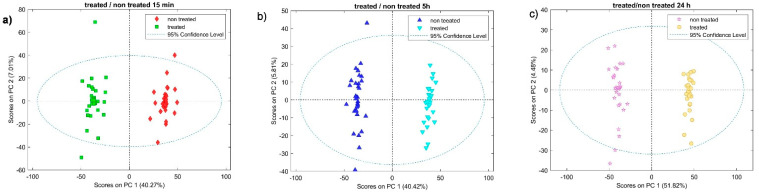
PCA score plots (PC1 vs. PC2) of mean-centered FTIR spectra for treated and non-treated samples after (**a**) 15 min, (**b**) 5 h, and (**c**) 24 h. The percentages in parentheses indicate the explained variance of each principal component. The dashed ellipse denotes the 95% confidence ellipse for the PCA model.

Two-dimensional PCA score plots (PC1 vs. PC2) constructed separately for each time point further highlighted clear discrimination between untreated and treated animals. PCA score plots are shown in Figure 2a–c, corresponding to 15 min (Figure 2a), 5 h (Figure 2b), and 24 h (Figure 2c). PCA score plots were used to visualize clustering. Clusters representing BPC 157 groups were displaced relative to their corresponding controls, and the direction of displacement was consistent with the trend seen in the global 3D model.

SVMDA classification of FTIR spectra into six classes (control vs. BPC 157 at 15 min, 5 h, and 24 h) showed excellent performance. The SVMDA model was trained on the same preprocessed experimental FTIR spectra used for the PCA; therefore, the classification performance reflects the magnitude and consistency of the spectral differences identified by the PCA separation and supported by the corresponding loading plots and Student’s *t*-test results. As summarized in Table 1, the classification report/per-class metrics (CV) shows high cross-validated true-positive rates across all six classes, while false-positive and false-negative rates, as well as overall error rates, remain low. The confusion matrix (CV) (lower part of Table 1) confirms this pattern at the level of individual spectra: the vast majority of observations fall on the main diagonal, with only a few misclassified spectra, mostly between neighboring time-adjacent classes. Together, the classification report and confusion matrix demonstrate that the FTIR signatures captured by the SVMDA model are robust enough to support reliable automatic discrimination between saline- and BPC 157-treated aortic segments at all examined time points.

### 2.2. Enhanced Amide I and II Bands in BPC 157-Treated Aortas

**Figure 3:**
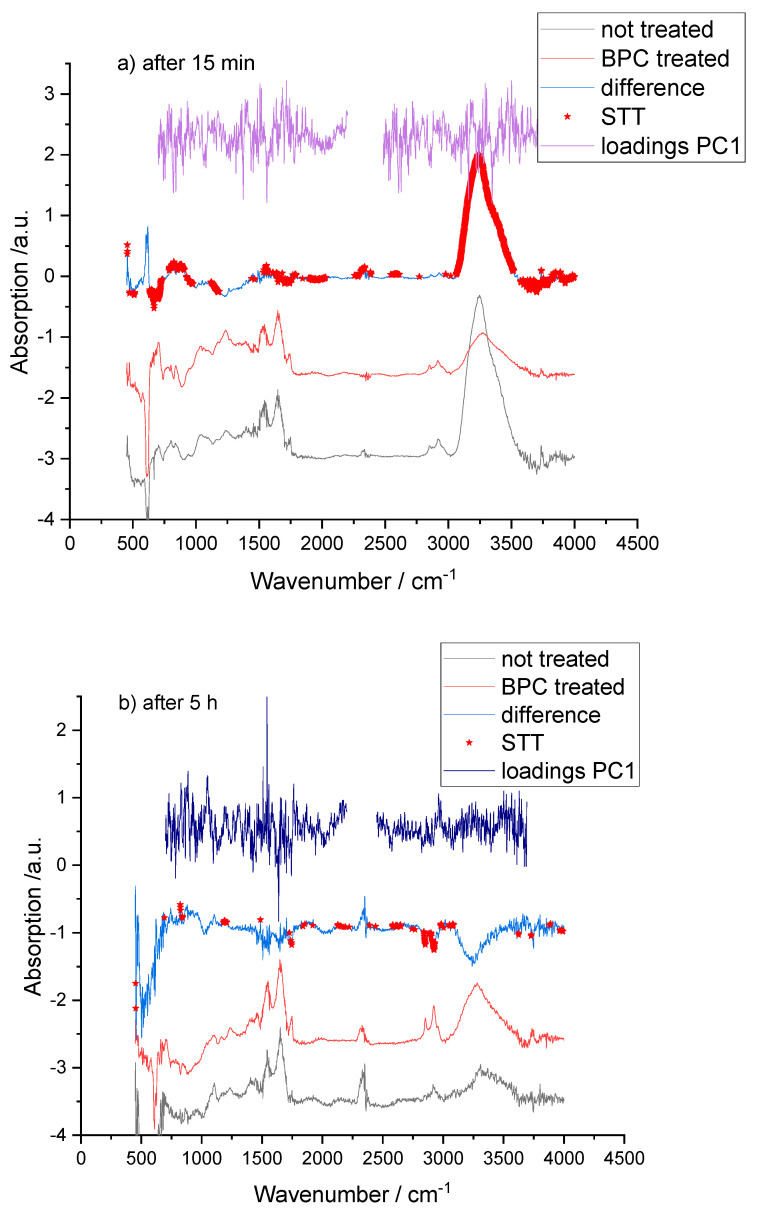

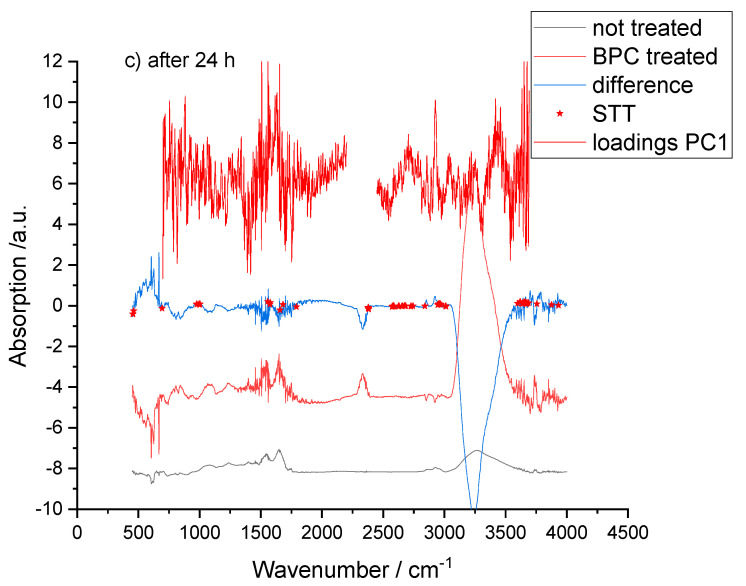
Mean FTIR spectra, difference spectra with added red stars for wavenumbers where Student’s *t* test shows significant differences (*p* < 0.05) and PC loadings highlighting significant treatment-related band changes (amide I, amide II, GAG- and lipid-associated regions) at 15 min (**a**), 5 h (**b**), and 24 h (**c**).

Separation between treated and non-treated samples occurs primarily along PC1 (40.27%, 40.42%, and 51.82% explained variance for 15 min, 5 h, and 24 h, respectively), while PC2 explains 7.01%, 5.81%, and 4.48% (Figure 2). PCA score plots (Figure 2a–c) show a clear multivariate separation between saline- and BPC 157-treated aortic spectra across the examined time points, indicating that treatment- and time-dependent differences are present at the level of the overall spectral profile. The corresponding PCA loading plots (Figure 3) identify the specific wavenumber regions that contribute most strongly to PC1/PC2 and therefore drive the observed clustering, providing an interpretable link between multivariate separation and FTIR band assignments. Consistently, Student’s *t*-test (STT) map highlights statistically different wavenumbers between groups that overlap with the high-absolute-loading regions, demonstrating concordance between univariate significance and the multivariate drivers of separation. Together, the agreement between scores (group separation), loadings (spectral features responsible for separation), and the STT map (significant wavenumber differences) supports the robustness of the identified FTIR spectral signatures and strengthens their biochemical interpretation in the context of vascular tissue composition and remodeling.

On difference spectra (BPC 157-treated minus control), red stars indicate wavenumbers where STT showed significant differences (*p* < 0.05). PC1 loading plots with their maximum values, combined with STT results, revealed that the most prominent treatment-related changes occurred in the amide I (~1660 cm^−1^) and amide II (~1540 cm^−1^) regions (Figure 3).

At the 15 min and 24 h time points, intensities at these bands were significantly higher in BPC 157-treated aortas than in saline controls (Figure 3a,c). At 5 h, the changes in amide I and II were smaller and did not individually meet the predefined significance threshold, but their direction and relative magnitude were consistent with those at the early and late time points (Figure 3b).

In vascular tissue, amide I and II bands arise predominantly from collagen- and elastin-rich ECM components, as well as from proteoglycan core proteins [48,51,53,55,56]. A shift toward higher intensity in these regions is consistent with a higher relative contribution of protein-associated bands and compatible with an increased contribution of collagen to the overall ECM spectral signature a change in secondary structure toward a more ordered, fibrillar state. In the present model, the rapid enhancement of amide I and II signals within 15 min of BPC 157 administration suggests that the peptide induces early reorganization and stabilization of the aortic ECM rather than changes that rely solely on de novo protein synthesis over many hours or days.

### 2.3. Collagen, Proteoglycan, and Glycosaminoglycan (GAG)-Associated Bands

Additional significant differences between treated and control spectra were observed in spectral intervals associated with collagen-specific CH_2_ modes, proteoglycans, and GAGs. At 15 min, the band near 1450–1456 cm^−1^, assigned to asymmetric CH_2_ bending in type I collagen, was significantly more intense in BPC 157-treated aortas than in controls (Figure 3a). This finding is compatible with a higher relative contribution of collagen-associated vibrations to the overall ECM spectral signature and mirrors the amide I/II enhancements [48,56].

In the lower wave number region, bands within 1170–1102 cm^−1^ and 955–926 cm^−1^ also discriminated treated from control samples, with higher intensities in the BPC 157 group (Figure 3a). These bands are generally attributed to sulfate and carbohydrate vibrations in GAG chains and to aggrecan and related proteoglycans [48,57]. Their increase indicates remodeling of proteoglycan/GAG components within the aortic wall. Proteoglycans and GAGs are key determinants of ECM hydration, viscoelastic properties, and interactions with lipoproteins, and their preservation or enhancement may help maintain vascular compliance and barrier integrity after adrenalectomy.

At the 5 h time point, although differences in amide I and II bands were less pronounced, a significant change was detected around 1204 cm^−1^, assigned to amide III and CH_2_ wagging vibrations of the collagen backbone and proline side chains [48]. This suggests subtle, ongoing modifications in collagen conformation during the intermediate phase after BPC 157 treatment, even when gross differences in total protein content are less evident (Figure 3b).

### 2.4. Lipid-Related Bands and Membrane Preservation

High-intensity bands around 2850 cm^−1^ and 2920–2930 cm^−1^, corresponding to symmetric and asymmetric stretching of CH_2_ and CH_3_ groups in lipid acyl chains, also contributed to the separation between control and BPC 157-treated spectra [1,47,49]. In general, BPC 157-treated aortas exhibited altered intensities in these lipid-associated bands compared with saline controls, consistent with preservation or reorganization of membrane and lipid structures in the vascular wall.

## 3. Discussion

For the vascular recovery as the major point of the BPC 157 therapy, the present FTIR spectroscopy study after unilateral adrenalectomy, considerably extends the recent highlight in healthy rats, the rapid peptide-induced molecular changes in rat blood vessels, particularly in lipid content and protein secondary structure as a background for the vascular function under severe stress [1]. In this manner, FTIR reveals molecular signatures of adrenalectomy-induced vascular injury (i.e., ECM degradation, lipid peroxidation, and loss of GAG content, leading to impaired vessel viscoelasticity and barrier function), which are mitigated by BPC 157. In the adrenalectomy model, BPC 157 appears to activate this protective program within the vascular wall, enabling the circulation to withstand the abrupt hemodynamic and endocrine perturbation caused by the removal of one adrenal gland. Within 15 min of peptide administration, spectra already show enhanced amide I and II bands and collagen- and GAG-related features, suggesting early ECM-related remodeling at a molecular level. These changes persist or re-emerge at 24 h, suggesting both an immediate and a sustained phase of matrix adaptation. On the other hand, in general, with respect to vascular recovery after unilateral adrenalectomy, the therapy effect on these molecular changes could be indicative of recovery. This could reflect the at least partial substitution of the function of the removed adrenal gland. Likewise, it could reflect the recovery of the remaining adrenal gland’s function, which would otherwise remain dysfunctional for a considerable period [58,59].

In addition, BPC 157-treated aortas showed higher intensities of amide I components commonly attributed to collagen and elastin, as well as the intensity of CH_2_ bending modes specific to collagen, band changes in the 1660, 1540, and 1450 cm^−1^ regions directly coincide with rapid restoration of physiological blood pressure gradients, noted in the recovery of the occlusion/occlusion-like syndromes. Thus, from a mechanistic standpoint, a more resilient collagen–elastin scaffold, it may be the strengthened collagen–elastin network, the more efficient transmission of pulsatile energy, and better matching between central and peripheral resistance given the reorganized ECM supports. Furthermore, FTIR signals in the 1170–1102 cm^−1^ and 955–926 cm^−1^ ranges, as well as specific amide I and II components, are sensitive to the presence and conformation of proteoglycan and GAG [48,53], contributing to the osmotic, viscoelastic, and barrier properties of the vascular ECM. Also, the increased GAG-related bands observed in BPC 157-treated aortas consequent to the peptide preserving or restoring proteoglycan/GAG content in the vascular wall during the acute stress of adrenalectomy, may have a broader significance (i.e., given the recovering effect on the lesions in counteraction of the severe vascular and multiorgan failure in various occlusion/occlusion-like syndromes [29,30,31,32,33]). As such, this may also be related to the evidence that in cartilage and other GAG-rich tissues, changes in these bands correlate with hydration state, compressive stiffness, and degenerative processes [48,53,60]. On the other hand, the spectral profile of BPC 157-treated aortas is congruent with a vessel wall that is less thrombogenic, higher amide I and II band intensities consistent with preserved collagen and elastin-related spectral features, preserved proteoglycans and GAGs, and stabilized lipids together suggest an endothelium-supported ECM that resists denudation and exposure of pro-thrombotic subendothelial components. As pointed out with BPC 157 therapy against various occlusion/occlusion-like syndromes, vascular recovery, collateral rescuing pathways activation (i.e., azygos vein direct blood flow delivery) occur along with counteraction of severe vascular and multiorgan failure, both arterial and venous thrombosis reversed, and Virchow triad circumstances annihilated [29,30,31,32,33]. Finally, changes in CH_2_ and CH_3_ stretching bands at ~2850 and ~2920–2930 cm^−1^ reflect alterations in lipid chain content, ordering, and saturation [1,47,49]. The differences between control and BPC 157-treated aortic spectra in these regions, together with the normalization of MDA levels by BPC 157 noted in many occlusion/occlusion-like syndromes recovery [29,30,31,32,33] and other studies (for review see, i.e., [2,38,42,43]), indicate that the peptide counteracts lipid peroxidation and preserves membrane architecture. So, the attenuation of lipid peroxidation observed in FTIR spectra corresponds to BPC 157’s well-documented reduction in oxidative stress markers. Similar FTIR-detected lipid modifications have been reported in brain and heart tissues in relation to ischemia, reperfusion, and neurodegeneration [52,55,56,61].

Finally, providing overall PCA clustering showing the clear separation of BPC 157-treated and control aortic spectra at all time points mirrors the PCA space that can be viewed as an abstract “fingerprint” of the vascular phenotype. Spectra clustering with the BPC 157 corresponds to animals with recovered severe vascular and multiorgan failure, attenuated/eliminated blood pressure disturbances, and thrombosis in counteraction of various occlusion/occlusion-like syndromes; therefore, it is relevant for recovery course after unilateral adrenalectomy, as well as occlusion/occlusion-like syndrome [29,30,31,32,33], in general. There is also interaction with various molecular pathways [34,35,36,37,38,39,40,41,42,43,44,45,46]. Thus, viewed in this integrated framework, FTIR spectroscopy, instead of hemodynamics and histology, adds a molecular resolution layer that at least partly further explains the beneficial effect afforded by BPC 157. Also, it might be that FTIR spectroscopy provides a sensitive molecular platform for evaluating vascular injury and therapeutic recovery in endocrine-related vascular fragility. This may be particularly so given the acknowledged congruence between rat and human unilateral adrenalectomy [62,63,64,65], as rat adrenalectomy models reliably reflect human adrenalectomy physiology and compensatory mechanisms [62,63,64,65]. Also, ng-regimens via the intragastric route occur as a conceptual follow-up of the cytoprotection concept, a cytoprotection mediator native and stable in human gastric juice, suited for further use in therapy [2].

## 4. Materials and Methods

### 4.1. Animals

The study was conducted with appropriately randomized male albino Wistar rats, 12–16 weeks of age, with 280 g body weight, self-breeding in the Department of Pharmacology, Faculty of Medicine, Zagreb, Croatia. The facility for animals was registered by the Veterinary Directorate (Reg. No.: HR-POK-007). Laboratory rats were acclimatized for five days and assigned identification numbers prior to allocation. Randomization was performed using a computer-generated random number sequence with block randomization to ensure equal group sizes (*n* = 6 animals per group per time point) across all treatments and postoperative intervals. The laboratory animals were housed in polycarbonate (PC) cages in conventional laboratory conditions at 20–24 °C, relative humidity of 40–70%, and noise level of 60 dB. The cages were identified with the dates, study number, group, dose, number, and sex of each animal. Twelve-hour daylight was provided by fluorescent lighting. They received standard nutrition (pelleted feed) and fresh water by free access (ad libitum) in accordance with Good Laboratory Practice (GLP). The care of the animals was in accordance with the standard operating procedures of the facility for pharmacological animals and the European Convention for the Protection of Vertebrate Animals Used for Experimental and Other Scientific Purposes (ETS 123). This research was approved by the local Ethics Committee (School of Medicine Zagreb, University of Zagreb; case number 380-59-10106-17-100/290; approval date: 30 October 2017) and by the Directorate of Veterinary (UP/I-322-01/15-01/22). The ethical principles of the study were in accordance with the European Directive 2010/63/EU, the Act on Amendments to the Animal Protection Act (Official Gazette 37/13), the Animal Protection Act (Official Gazette 135/06), the Ordinance on the Protection of Animals Used for Scientific Purposes (Official Gazette 55/13), the recommendations of the Federation of European Laboratory Associations for Animal Science (FELASA), and the recommendations of the Ethics Committee of the Faculty of Medicine, University of Zagreb. The experiments were evaluated by an independent observer who was blinded to the treatment allocation.

A priori power analysis was performed for a representative primary outcome, assuming a two-sided significance level of 0.05 and a statistical power of 80%. Based on effect sizes observed in previous studies using the same experimental model, a large, standardized effect size (Cohen’s d = 1.2) was assumed. Under these conditions, a minimum of 6 animals per group was required to detect a statistically significant difference between groups. Accordingly, group size was set at *n* = 6 animals per group per time point.

### 4.2. Drugs

Stable gastric pentadecapeptide BPC 157 (GEPPPGKPADDAGLV, molecular weight 1419; Diagen, Ljubljana, Slovenia), a partial sequence of the human gastric juice protein BPC, which is freely soluble in water at pH 7.0 and in saline, was prepared as a peptide with 99% high-performance liquid chromatography (HPLC) purity, with 1-des-Gly peptide being the main impurity. The BPC 157 dose and application regimens (10 ng/kg given as an intragastric administration) were as described previously (i.e., without the use of a carrier or peptidase inhibitor) (for review see, i.e., [29,30,31,32,33]).

### 4.3. Animal Model and Experimental Design

Unilateral adrenalectomy (left adrenal gland) was performed under general anesthesia induced by intraperitoneal thiopental (40 mg/kg) (Rotexmedica GmbH/Panpharma GmbH, Trittau, Germany) and diazepam (10 mg/kg) (Krka, Novo Mesto, Slovenia). The procedure was carried out via a classic dorsal approach. After adrenalectomy, animals were randomly allocated to receive either intragastric saline (5 mL/kg) or BPC 157 (10 ng/kg). Sacrifice was performed at 15 min, 5 h, or 24 h after surgery [1].

### 4.4. Aortic Sampling for FTIR Spectroscopy

Abdominal aorta segments (0.5–1 cm in length) were excised from rats at each time point (15 min, 5 h, 24 h) after unilateral adrenalectomy. Samples were embedded in a drop of distilled water and snap frozen. For each animal, on average, 5 cryosections 60 µm thick were cut and placed on silicon windows (Pike Technologies, Fitchburg, WI, USA). The exact number of cut samples depends on the sample size. Excess water was removed by vacuum dehydration to minimize interference in the mid-infrared region, as FTIR spectra are highly sensitive to water absorption. After dehydration, it is assumed that all processes induced by the treatment have ceased.

### 4.5. FTIR Spectral Acquisition and Preprocessing

Vibrational spectra were recorded using a PerkinElmer Spectrum GX spectrometer (Waltham, MA, USA) equipped with a mercury-cadmium-telluride (MCT) detector. First, silicon wafers without tissue were measured to obtain background spectra. Background spectra were collected with 1000 scans, averaged, and automatically subtracted from the subsequent sample spectra. Each tissue spectrum is made by 400 averaged scans, which took approximately 6 min to acquire per sample. The spectral data were collected in the range of 450 to 4000 cm^−1^ with a spectral resolution of 4 cm^−1^ [1]. In this study, the whole spectrum was used for analysis.

All spectra were subjected to baseline correction and normalization. The band at 1646 cm^−1^, which corresponds to the amide I peak, was used as an internal reference. To perform baseline correction, we identified low-intensity anchor points across the spectrum, interpolated straight lines between them, and subtracted the resulting baseline from the raw spectra. This preprocessing step is crucial for minimizing variations caused by sample thickness, recording conditions, and other physical and chemical factors. It is a standard procedure in FTIR analyses of biological tissues [47,49,50,55,56,57,61].

### 4.6. Multivariate and Univariate Spectral Analysis

Preprocessed spectra were exported to MATLAB (R2010b, MathWorks, Natick, MA, USA) and analyzed using PLS_Toolbox 7.0 (Eigenvector Research, Manson, WA, USA). Two algorithms from this software, PCA and SVMDA, were used in this study. Principal component analysis (PCA) was performed in PLS_Toolbox (MATLAB) on mean-centered FTIR spectra. Score plots (PC1 vs. PC2) were used to visualize clustering between treated and non-treated samples. In contrast, loading plots were examined to identify the spectral regions (wavenumbers) contributing most to each principal component.

PCA loading plots were examined to identify the wavenumber regions contributing most to the principal components responsible for group separation, providing an interpretable link between multivariate patterns and FTIR band assignments.

To quantify class separation, support vector machine discriminant analysis (SVMDA) was applied. A six-class model was built for each combination of treatment (control or BPC 157) and time point (15 min, 5 h, or 24 h). Model performance was evaluated by 10-fold cross-validation, yielding confusion matrices, true positive and true negative rates, and misclassification indices.

In parallel, Student’s *t*-tests (STTs) were applied to compare the integrated intensities of selected absorption bands between control and BPC 157-treated aortas at each time point. For the 15 min and 24 h intervals, *p* < 0.02 was considered significant, whereas for the 5 h interval *p* < 0.05 was used. Band assignments were based on prior FTIR studies of vascular, cartilage, and other connective tissues and are presented in Table 2, derived from the data presented in references [1,47,48,51,53,55,56,57,61].

### 4.7. Band Assignments

Band assignments important to this paper are listed in Table 2.

### 4.8. Summary of Leading FTIR Bands and Their Biological Meaning

A concise summary of the principal spectral markers used to interpret BPC 157 effects on the aortic wall is provided in Table 3.

## 5. Conclusions

FTIR spectroscopy reveals that unilateral adrenalectomy produces distinct molecular signatures of vascular fragility—characterized by reduced amide I/II intensity, diminished collagen- and GAG-associated bands, and increased lipid peroxidation-related features. FTIR spectroscopy of abdominal aorta reveals that BPC 157 induces rapid (15 min) and sustained (5–24 h) remodeling of the vascular wall after unilateral adrenalectomy in rats. The peptide is associated with higher intensities of amide I/II and collagen-, proteoglycan-, GAG-, and lipid-associated bands, consistent with early ECM stabilization/remodeling and membrane preservation. These molecular signatures align with the recovery course after unilateral adrenalectomy, as well as occlusion/occlusion-like syndrome, in general.

## Figures and Tables

**Table 1:** Cross-validation confusion matrix and classification report for six classes (Class 1: 15 min BPC 157 treated; Class 2: 15 min not treated (saline); Class 3: 5 h BPC 157 treated; Class 4: 5 h not treated (saline); Class 5: 24 h BPC 157 treated; Class 6: 24 h not treated (saline)).

**Classification Report/Per-Class Metrics (CV)**								
**Class**	**TPR**	**FPR**	**TNR**	**FNR**	**N**	**Err**	**P**	**F1**
Non treated 15 min	0.83871	0.01282	0.98718	0.16129	31	0.03743	0.92857	0.88136
Treated 15 min	0.90323	0.00000	1.00000	0.09677	31	0.01604	1.00000	0.94915
Non treated 5 h	0.93939	0.02597	0.97403	0.06061	33	0.03209	0.88571	0.91176
Treated 5 h	0.96774	0.01923	0.98077	0.03226	31	0.02139	0.90909	0.93750
Non treated 24 h	0.96774	0.03205	0.96795	0.03226	31	0.03209	0.85714	0.90909
Treated 24 h	0.76667	0.03185	0.96815	0.23333	30	0.06417	0.82143	0.79310
**Confusion Matrix (CV)**								
**Predicted/Actual**			**Class 1**	**Class 2**	**Class 3**	**Class 4**	**Class 5**	**Class 6**
Non treated 15 min			26	1	0	0	0	1
Treated 15 min			0	28	0	0	0	0
Non treated 5 h			1	1	31	0	0	2
Treated 5 h			0	0	2	30	0	1
Non treated 24 h			2	0	0	0	30	3
Treated 24 h			2	1	0	1	1	23
Predicted as Unassigned			0	0	0	0	0	0

TPR = true positive rate (sensitivity/recall); FPR = false positive rate; TNR = true negative rate (specificity); FNR = false negative rate; N = number of samples in the class; Err = misclassification rate (1 − accuracy); P = precision (positive predictive value); F1 = F1-score.

**Table 2:** Standard band assignments are based on established FTIR literature and supported by primary FTIR studies of aortic tissue [51].

Wavenumber (cm^−1^)	Assignment	References
~1660	Amide I—C=O stretching; dominated by type I collagen and elastin	[48,51,53,56]
~1654–1655	Collagen- and elastin-specific Amide I components in aortic and valvular tissues	[51,55,56]
~1640	Amide I contribution from proteoglycans	[48,55]
~1540–1545	Amide II—C–N stretching and N–H bending; strong proteoglycan contribution	[48,55]
~1450–1456	CH_2_ bending vibrations characteristic of type I collagen	[48,54]
~1204	Amide III—CH_2_ wagging from collagen backbone and proline side chains	[48]
1170–1102 and 955–926	GAG-related bands: sulfate, carbohydrate and aggrecan-associated modes	[48,57]
~2920 and ~2850	Asymmetric and symmetric CH stretching (CH_2_/CH_3_) of saturated fatty acids; lipid content and ordering	[1,47,61]

**Table 3:** Principal FTIR bands used in this study and their biological interpretation in the context of BPC 157 action after unilateral adrenalectomy [1,47,48,51,53,55,56,57,61].

Wavenumber (cm^−1^)	Band/Assignment	Change with BPC 157
~1660	Amide I (collagen, elastin)	Increased intensity, consistent with enhanced collagen–elastin–associated spectral contribution
~1540–1545	Amide II (proteins, proteoglycans)	Increased intensity, consistent with enhanced protein content and proteoglycan integrity
~1450–1456	CH_2_ bending (type I collagen)	Increased intensity at 15 min; suggests a strengthened collagen network
~1204	Amide III/CH_2_ wagging	Significant change at 5 h; suggests ongoing collagen-related conformational changes
1170–1102	GAG-related sulfate/carbohydrate modes	Higher intensity in treated aortas is consistent with preserved or restored GAG-associated spectral signatures
955–926	GAG- and aggrecan-associated modes	Increased intensity, supportive of barrier and hydration properties of ECM
2920, 2850	CH stretching in lipid acyl chains	Altered intensities compatible with reduced lipid oxidative modification related features and membrane preservation

## Data Availability

The original contributions presented in the study are included in the article, further inquiries can be directed to the corresponding authors.
